# Society’s Attitude Toward Spousal Physical Abuse: Findings from the Philippines National Demographic and Health Survey, 2022

**DOI:** 10.1089/whr.2024.0052

**Published:** 2024-10-04

**Authors:** Wah Wah Myint, Roaa Aggad, Qiping Fan, Chimuanya Osuji, Heather R. Clark, E. Lisako Jones McKyer

**Affiliations:** ^1^ Center for Community Health and Aging, School of Public Health, Texas A&M University, College Station, Texas, USA.; ^2^ Department of Family and Community Medicine, King Abdulaziz University, Rabigh, KSA.; ^3^ Department of Public Health Sciences, Clemson University, Clemson, South Carolina, USA.; ^4^ Department of Health Behavior, School of Public Health, Texas A&M University, College Station, Texas, USA.; ^5^ Center for Health Equity and Evaluation Research, School of Public Health, Texas A&M University, College Station, Texas, USA.; ^6^ Department of Population & Community Health, University of North Texas, Fort Worth, Texas, USA.

**Keywords:** intimate partner violence, spousal physical abuse, Philippines, social determinants, health behavior

## Abstract

**Background:**

Societal attitude toward spousal physical abuse plays a crucial role in preventing violence against women. Yet, this public health issue has been insufficiently addressed. This study examines the relationship between the societal attitude toward spousal physical abuse and various social determinants.

**Methods:**

We used data from the 2022 Philippines’ National Demographic and Health Survey. The outcome variable was attitude toward spousal physical abuse. Covariates included women’s sociodemographic characteristics, experiences of witnessing their father’s abusive behavior, intimate partner violence (IPV), and controlling behavior. Partner-related variables (age, educational level, employment status, and alcohol consumption behavior) were also considered. Descriptive and logistic regression analyses were performed to examine the associated factors of spousal physical abuse by using Stata 18.0.

**Results:**

Overall, 1,920 (9%) of 19,228 women reported that spousal physical abuse is justifiable in at least one of the presented scenarios. Women IPV survivors (adjusted Odds Ratio [aOR] = 1.35, 95% confidence interval [95% CI] = 1.06–1.73) and those who experienced controlling behavior by their partners (aOR = 1.77, 95% CI = 1.45–2.15) were more likely to accept spousal physical abuse than their counterparts. Conversely, women with a higher decision-making score were less likely to accept spousal physical abuse than those who had a lower score (aOR = 0.74, 95% CI = 0.56–0.98).

**Conclusions:**

Finding suggests that women’s attitudes toward spousal physical abuse are significantly influenced by their experience of IPV. Future health research, programs, and policies should address individual, interpersonal, and systemic-level risk factors that profoundly impact women’s health.

## Introduction

### Spousal physical abuse: Global context

Spousal physical abuse, a form of interpersonal violence and a prevalent issue in contemporary society, represents a global public health crisis and a violation of human rights with multiple consequences.^1–4^ Spousal physical abuse is often employed synonymously with battering, domestic violence, violence against females/women, intimate partner violence (IPV), intimate partner aggression, and intimate partner coercion.^5^ Experiencing IPV is linked to a range of negative psychological and physical outcomes, including stress, poor physical and mental health, and adverse birth effects, all of which impact both the family unit and the broader society.^6–10^ Studies indicate that 16.5%–22.8% of women have experienced physical harm from aggression in their lifetimes.^11^ Walker-Descartes et al. highlighted that while the psychological impact of such violence can be subtle, it is as debilitating as physical harm.^12^ Furthermore, IPV survivors often endure multiple forms of violence—physical, psychological, and sexual—which can be recurrent.^1^

Societal attitudes toward IPV against women contribute to the significant challenges faced by survivors. Globally, cultural attitudes toward such behavior vary.^13^ While some societies condemn it, others may justify wife-beating due to entrenched social norms or customs.^13–15^ Schuster and colleagues found that acceptance of spousal physical abuse among Jordanian adolescents varied widely.^16^ Similarly, a study in Syria showed that <17% of future health care professionals rejected the justification of spousal physical abuse.^17^ In countries such as Chile and Brazil, some view physical violence as a means of disciplining wives,^13^ while in India, despite the prevalence of sexual violence within marriage, rape is often not recognized as a crime.^3^ Also, the study done by Edwards revealed that victims with lower self-esteem are more likely to remain silent in abusive relationships,^18^ and reasons reported by victims in some studies included societal perception, religious beliefs, and cultural barriers.^19–21^ A study among urban African American adolescents found that only 52% disclosed their experiences of victimization,^6^ while another study found that factors such as religious beliefs and cultural norms can impede victims from leaving abusive relationships or seeking help.^21^ The societal perception of IPV and cultural norms make challenges for disclosure for many survivors.

Intergenerational violence, hierarchical gender relations, and socioeconomic factors are also significant for the violence perpetration or being victimized in a person’s intimate relationship.^22–26^ The women who witnessed the intergenerational violence were more likely to experience IPV than those who did not.^22,23^ Hierarchical gender relations predispose women to aggression^24^ and factors like age are also crucial determinants for being abused.^25^ Furthermore, Antai et al.^26^ and Begum et al.^24^ noted that the socioeconomic inequalities make women more susceptible to being abused and manipulated by their husbands.^,^

### Spousal physical abuse: The Philippines’ context

Spousal physical abuse occurs across various countries and cultural settings, with Southeast Asia reporting high incidents of partner violence against women.^27,28^ The Philippines, home to nearly 175 ethnolinguistic groups with unique cultural identities and practices, has made progress in women’s rights and protection.^29^ However, in this archipelago, one in four women has experienced gender-based violence.^30^ Despite having legal frameworks such as the Philippines’ Republic Act 9262 and the Anti-Violence Against Women and Their Children Act of 2004, women in the Philippines continue to face challenges due to a patriarchal society that reinforces male dominance in familial and social structures.^31–33^ The Philippines also grapples with health inequalities that challenge significant policy change, especially for women.^32^

This study explores factors associated with the societal attitude toward spousal physical abuse among Filipino women. This research intends to respond to the following research questions.

1. What are the factors influencing societal attitudes toward spousal physical abuse?
2. Are there linkages between the different types of experienced abuse and attitudes toward spousal physical abuse?
3. How does the score in decision-making impact attitude toward spousal physical abuse?
4. Is there an association between women’s awareness of the Barangay Violence Against Women (VAW) law and their acceptance of spousal physical abuse?
5. Is there an association between experiencing control by a partner and the acceptance of spousal physical abuse?

## Methods

### Data source

This study used cross-sectional deidentified data from the Philippines’ National Demographic and Health Survey (P-NDHS), 2022,^34^ which has been reviewed and approved by the Inner-City Fund’s Institutional Review Board.^35^ A total of 27,821 women participated in the P-NDHS. For this study, we included a married women sample (*n* = 19,228), who met the eligibility criteria and responded to the domestic violence module.^34,35^ The eligibility criteria were (1) one woman per household, (2) if the privacy of the respondent was obtained, and (3) the woman is either currently married/living with a man or formerly married/lived with a man.^35^ The domestic violence module has questions related to women’s experiences of different types of IPV, controlling behaviors by their partners, disclosure, and help-seeking behavior.^35^ This study used secondary de-identifiable data, which are publicly available on the Demographic and Health Surveys website.^36^ The data were collected using two-stage random sampling.^34^

### Variables

#### Outcome variable

The outcome variable for this study was respondents’ attitude toward spousal physical abuse (do not accept spousal physical abuse = 0, accept spousal physical abuse = 1). This is a composite variable created from five scenarios of the survey: accept that wife-beating is justified if a woman goes out without telling her husband/partner (No = 0, Yes = 1), neglects children (No = 0, Yes = 1), argues with husband/partner (No = 0, Yes = 1), denies sex (No = 0, Yes = 1), and burns the food (No = 0, Yes = 1). For this article, the term “spousal physical abuse” represents the term “wife-beating,” which is used in the original survey.

#### Respondent’s sociodemographic variables

Covariates included sociodemographic characteristics of female participants respondents’ age in 5-year groups (15–19, 20–24, 25–29, 30–34, 35–39, 40–44, 45–49), place of residency (urban, rural), education level (no education, primary, secondary, higher), wealth quintiles (poorest, poorer, middle, richer, richest), marital status (never married, ever married), and current employment (No = 0, Yes = 1).

#### Covariates

We included decision-making scores (score 0, score 1, score 2, score 3, score 4). The decision-making score is a composite variable created from four decision-making scenarios: person making decision on the respondent’s health care, on large household purchase, visits to families/relatives, and husband/partner’s earnings. We also included witnessing the father’s abusive behavior (No = 0, Yes = 1), awareness of Barangay VAW law (No = 0, Yes = 1), experienced different types of violence (no IPV = 0, physical IPV = 1, sexual IPV = 2, emotional IPV = 3), and experienced controlling behavior (No = 0 for no experience of controlling behavior, and Yes = 1 for experience of controlling behavior). The controlling behavior is a composite variable from five scenarios: Husband/partner is jealous if respondent talks with other men, accuses respondent of unfaithfulness, does not permit respondent to meet female friends, tries to limit respondent’s contact with her family, and insists on knowing where the respondent is.

#### Partner’s sociodemographic variables

Partner’s sociodemographic variables were also included and consisted of age (in 5-year groups), education level (no education, primary, secondary, and higher), employment (No = 0, Yes = 1), and alcohol-drinking behavior (No = 0, Yes = 1).

### Analyses

Descriptive statistics of outcome, respondents’ sociodemographics, covariates, and the husbands’ sociodemographic variables were conducted to have a better understanding of the included variables. The bivariate analyses were performed to determine the relationship between the spousal physical abuse and all the variables listed above. We applied multivariable logistic regression (LR) to predict the relationship between the outcome variable, that is, spousal physical abuse, and other covariates. For the multivariable LR, we planned to include all the respondents’ sociodemographic variables (respondents’ age, place of residence, education level, wealth quintiles, and current employment status) regardless of their significance level. However, for other variables, we planned to consider variables that showed significant association in the bivariate analysis. The significance level considered for bivariate and multivariable analyses was *p* < 0.05. All analyses applied survey weights using Stata 18.0.^37^

## Results

### Results from univariate analysis

Table 1 shows the characteristics of women who participated in the domestic violence module. Among them, the highest percentage were the 15–19 age group (19%, *n* = 3,021) and the 20–24 age group (17%, *n* = 2,776). The respondents from urban areas were slightly more than the rural respondents (56%, *n* = 11,485 vs. 44%, *n* = 7,743). More than half (53%, *n* = 9,622) of respondents had secondary education. Among the women respondents, 9% (*n* = 1,920) reported they accepted wife-beating in a given scenario, 18% (*n* = 2,721) experienced any type of IPV at least once in their lifetime, and 36% (*n* = 5,262) reported to have been controlled by their partners, although 88% (*n* = 17,033) reported they were aware of Barangay VAW law and 62% (*n* = 11,850) reported they made a decision either by themselves or with their partners. Tables 1 and 2 present the prevalence of included variables including sampled women and their husband/partner's sociodemographic characteristics and other covariates.

**Table 1.** Sociodemographic Characteristics of Sampled Women (*n* = 19,228)

Sociodemographic characteristics	*n*	%
Attitude toward spousal physical abuse		
Accept that spousal physical abuse is not justified	17,308	91
Accept that spousal physical abuse is justified	1,920	9
Total	19,228	100
Respondents’ age groups		
15–19	3,021	19
20–24	2,776	17
25–29	2,890	14
30–34	3,121	14
35–39	2,738	12
40–44	2,512	12
45–49	2,170	12
Total	19,228	100
Place of residence		
Urban	7,743	56
Rural	11,485	44
Total	19,228	100
Education level		
No education	212	1
Primary	2,290	9
Secondary	9,622	53
Higher	7,104	38
Total	19,228	100
Wealth quintiles		
Poorest	5,067	16
Poorer	4,231	19
Middle	3,561	20
Richer	3,194	22
Richest	3,175	23
Total	19,228	100
Marital status		
Never married	6,322	41
Ever married	12,906	59
Total	19,228	100
Current employment		
No	10,583	55
Yes	8,645	45
Total	19,228	100
Witnessing father’s abusive behavior		
No	16,478	87
Yes	2,750	13
Total	19,228	100
Decision-making score (either self or self and husband/partner)		
No	7,338	46
Score = 1	201	1
Score = 2	537	2
Score = 3	1,602	7
Score = 4	9,550	44
Total	19,228	100
Awareness of violence against women’s law		
No	2,195	12
Yes	17,033	88
Total	19,228	100
Experienced controlling behavior		
No	9,324	64
Yes	5,262	36
Total	14,586	100
Experienced different types of intimate partner violence (IPV)		
No	11,865	83
Physical IPV	260	2
Sexual IPV	93	1
Emotional IPV	2,368	15
Total	14,568	100

**Table 2.** Sociodemographic Characteristics of Respondent’s Husband/Partner

Sociodemographic characteristics	*n*	%
Husband/partner’s age groups		
15–19	34	1
20–24	643	3
25–29	1,510	7
30–34	2,394	10
35–39	2,397	10
40–44	2,211	10
45–49	10,039	60
Total	19,288	100
Husband/partner’s educational level		
No education	204	1
Primary	1,799	11
Secondary	6,277	52
Higher	4,000	36
Total	12,280	100
Husband/partner’s employment		
No	703	6
Yes	11,577	94
Total	12,280	100
Husband/partner’s alcohol-drinking behavior		
No	6,999	46
Yes	7,587	54
Total	14,586	100

### Results from bivariate analysis

Bivariate analyses revealed that respondents’ sociodemographic characteristics (age groups, place of residency, education level, wealth quintiles, marital status, current employment status), and covariates (witnessing father’s abusive behavior, awareness of violence against women’s law, having been controlled by partner, and experience of IPV, partner’s education level) were positively associated with attitude toward spousal physical abuse (Table 3). Contrarily, place of residence, marital status, and decision-making score, and partner’s variables (age groups, employment, alcohol-drinking behavior) did not affect the attitude toward spousal physical abuse.

**Table 3.** Bivariate Analysis: Attitude toward Spousal Physical Abuse

	Accept that spousal physical abuse is justified (yes)			
Sociodemographic characteristics	*n*	%	Total	*p*
Respondents’ age groups				
15–19	340	11.00	3,021	0.029
20–24	235	7.59	2,776	
25–29	274	8.95	2,890	
30–34	311	8.72	3,121	
35–39	272	9.60	2,738	
40–44	235	7.99	2,512	
45–49	253	10.30	2,170	
Total	1,920	9.21	19,228	
Place of residence				
Urban	683	9.00	7,743	0.066
Rural	1,237	10.00	11,485	
Total	1,920	9.00	19,228	
Education level				
No education	22	9.65	212	<0.001
Primary	325	12.80	2,290	
Secondary	1,065	10.30	9,622	
Higher	508	6.87	7,104	
Total	1,920	9.21	19,228	
Wealth quintiles				
Poorest	640	11.30	5,067	<0.001
Poorer	463	11.10	4,231	
Middle	363	9.55	3,561	
Richer	245	8.07	3,194	
Richest	209	6.88	3,175	
Total	1,920	9.21	19,228	
Marital status				
Never married	585	8.70	6,322	0.167
Ever married	1,335	9.57	12,906	
Total	1,920	9.21	19,228	
Current employment				
No	1,129	10.00	10,583	0.013
Yes	791	8.26	8,645	
Total	1,920	9.21	19,228	
Witnessing father’s abusive behavior				
No	1,573	9.00	16,478	0.024
Yes	347	11.00	2,750	
Total	1,920	9.00	19,228	
Decision-making score (either self or self and husband/partner)				
Others	689	9.00	7378	<0.001
Score = 1	38	18.00	201	
Score = 2	79	12.00	537	
Score = 3	211	12.00	1602	
Score = 4	903	8.00	9550	
Total	1,920	9.00	19,228	
Awareness of violence against women’s law				
No	297	13.00	2,195	<0.001
Yes	1,623	8.70	17,033	
Total	1,920	9.00	19,228	
Experienced controlling behavior				
No	778	7.00	9,324	<0.001
Yes	743	14.00	5,262	
Total	1,521	10.00	14,586	
Experienced different types of intimate partner violence (IPV)				
No	1,105	8.55	11,865	<0.001
Physical IPV	38	9.76	260	
Sexual IPV	21	21.40	93	
Emotional IPV	357	15.70	2,368	
Total	1,521	10.00	14,586	
Husband/partner’s age groups				
15–19	4	4.44	34	0.894
20–24	61	9.60	643	
25–29	159	8.87	1,510	
30–34	226	8.25	2,394	
35–39	239	9.64	2,397	
40–44	230	9.28	2,211	
45–49	1,001	9.31	10,039	
Total	1,920	9.21	19,228	
Husband/partner’s education level				
No education	21	10.50	204	<0.001
Primary	246	12.00	1,799	
Secondary	685	10.10	6,277	
Higher	313	7.41	4,000	
Total	1,265	9.38	12,280	
Husband/partner’s employment				
No	80	10.30	703	0.653
Yes	1,185	9.32	11,577	
Total	1,265	9.38	12,280	
Husband/partner’s alcohol drinking behavior				
No	707	9.15	6,999	0.152
Yes	814	10.30	7,587	
Total	1,521	9.74	14,586	

### Results from multivariable LR analysis

Overall, the sociodemographic variable showed an insignificant association with the attitude toward spousal physical abuse. For instance, the LR results showed women’s sociodemographic variables (women’s age groups, place of residence, education, and wealth quintiles, currently employed), and witnessing father’s abusive behavior did not demonstrate any significant association with the attitude toward spousal physical abuse (Table 4).

**Table 4.** Logistic Regression Results of Predictors of Attitude toward Spousal Physical Abuse

Outcome variable = attitude toward spousal physical abuse	
Sociodemographic characteristics	Odds ratio (95% CI)*
Respondents’ age groups	
15–19	
20–24	0.70 (0.32,1.53)
25–29	0.79 (0.38,1.65)
30–34	0.82 (0.39,1.71)
35–39	0.91 (0.42,1.95)
40–44	0.67 (0.32,1.37)
45–49	1.06 (0.49,2.29)
Place of residence	
Urban	
Rural	1.10 (0.87,1.4)
Education level	
No education	
Primary	1.08 (0.37,3.18)
Secondary	0.91 (0.31,2.69)
Higher	0.76 (0.24,2.35)
Wealth quintiles	
Poorest	
Poorer	0.97 (0.75,1.26)
Middle	0.84 (0.63,1.12)
Richer	0.71 (0.49,1.02)
Richest	1.07 (0.71,1.61)
Current employment	
No	
Yes	0.85 (0.68,1.06)
Decision-making score (either self or self and husband/partner)	
Others	
Score = 1	1.17 (0.51,2.69)
Score = 2	0.71 (0.31,1.61)
Score = 3	0.75 (0.35,1.59)
Score = 4	0.56 (0.27,1.16)
Witnessing father’s abusive behavior	
No	
Yes	0.85 (0.65,1.11)
Awareness of violence against women’s law	
No	
Yes	0.69 (0.48,0.99)*
Experienced controlling behavior	
No	
Yes	1.99 (1.6,2.44)***
Experienced different types of intimate partner violence (IPV)	
No IPV	
Physical IPV	0.88 (0.51,1.53)
Sexual IPV	2.57 (1.31,5.07)**
Emotional IPV	1.37 (1.04,1.79)*
Husband/partner’s education level	
No education	
Primary	1.22 (0.59,2.53)
Secondary	1.23 (0.57,2.67)
Higher	1.08 (0.47,2.47)
_cons	0.22 (0.06,0.88)*

_cons estimates baseline odds.

*p < 0.05; **p < 0.01; ***p < 0.001.

The first categories for each variable are reference categories.

CI, confidence interval.

However, there was a positive and significant association between experiencing IPVs and attitude toward spousal physical abuse. Among women who experienced different types of IPV, only those who had sexual (adjusted odds ratio [aOR] = 2.57, 95% confidence interval [95% CI] = 1.31–5.0) and emotional (aOR = 1.37, 95% CI = 1.04–1.79) violence were more likely to accept spousal physical abuse. However, women’s decision-making capabilities (either by themselves or joint decision-making with their partner) and acceptance of spousal physical abuse showed an insignificant association.

Contrarily, women’s awareness of Barangay VAW law showed a significant association with the acceptance of spousal physical abuse (aOR = 0.67, 95% CI = 0.47–0.99). There was a significant association between women’s experience of controlling behavior and acceptance of spousal physical abuse. Women who reported they experienced controlling behavior from their partners were twice more likely to accept spousal physical abuse (aOR = 1.99, 95% CI = 1.61–2.45) than those who did not.

## Discussion

The current study evaluated attitude toward spousal physical abuse among Filipino women. Our findings indicate significant correlations between the acceptance of spousal physical abuse and important factors such as IPV experience, controlling behavior by a husband/partner, and awareness of violence against women’s law. The overall findings addressed our first research questions regarding factors influencing the attitude toward spousal physical abuse.

Research in other contexts has shown that those who believe spousal physical abuse is justified in some circumstances are more likely to experience violence than those who do not believe spousal physical abuse is justified in any circumstance.^2^ Begum et al. also documented a higher prevalence of domestic violence against women who justified spousal physical abuse in India.^24^ Our study found that among women who experienced various types of IPV, only those who experienced sexual and emotional violence were more likely to accept spousal physical abuse. In other words, Filipino women who experienced sexual and emotional violence were more inclined to accept spousal physical abuse, possibly due to reduced self-confidence and increased fear. Further research is necessary to ascertain the mechanisms by which sexual and emotional violence predisposes women to spousal physical abuse.

Although our study did not find a significant association between physical violence and attitude toward spousal physical abuse, those who experienced physical IPV had the lowest odds of accepting it. This finding contradicts findings from Dikmen and Munevver’s^38^ study that revealed that the attitude toward spousal physical abuse was higher among those who had any type of IPV including physical IPV. Although our analyses did not measure a direct association with the attitude toward spousal physical abuse and potential serious effects like suicidal attempts, Brown and Seals^39^ revealed that 26% of suicidal cases had IPV issues. Hence, our finding regarding the association between physical IPV and attitude toward spousal physical abuse must be addressed immediately to prevent negative consequences such as suicidal attempts in this population. These findings address our second research question.

The present study did not show any significant association between attitude toward spousal physical abuse and women empowerment variables such as employment and decision-making authorities, which addressed our third research question. However, some studies have shown a strong association. Mercado et al. found that Filipino women experienced abuse by their husbands/partners regardless of their employment status.^40^ Also, lack of employment and justifying spousal physical abuse were significantly associated with economic abuse against women.^23^ Likewise, Atteraya and colleagues^41^ noted that spousal physical abuse diminished the decision-making autonomy. These results suggest that women may be more likely to experience controlling behavior and to endure spousal physical abuse if there is a socioeconomic disparity.

The current study’s finding that the women who were aware of Barangay VAW law were less likely to accept spousal physical abuse addressed our fourth research question. Given our finding that awareness of violence against women’s law has a protective effect against spousal physical abuse, policies and strategies that raise understanding of the law among Filipino women should be encouraged. This can impact women’s inclinations to seek assistance and lessen the detrimental effects of spousal physical abuse on their health. Future studies ought to examine the degree of success that the law has had in preventing female survivors of IPV as well as the barriers that prohibit them from utilizing the legal system to defend themselves.

Although our results failed to find significant associations between attitude toward wife-beating and decision-making authority of women, other sociodemographic factors appear to have influence. For instance, women in the older age groups (apart from the 40–49 age group), the wealthier groups (apart from the richest groups), and those with at least secondary education were less likely than those in the younger age groups (15–19), the uneducated groups, and the poorest groups to accept spousal physical abuse.

Some studies have shown that lower educational attainment of husbands/partners may encourage the use of violence to control their wives, irrespective of women’s educational levels. Puno et al. found female survivors of IPV with higher educational attainment than their partners were likely to accept spousal physical abuse.^25^ Thus, while a woman’s higher educational achievement should have shielded her from IPV, husband/partner’s lower educational attainment could still place her at risk of IPV victimization. Perhaps less-educated husbands/partners were more likely to hold traditional paternalistic views of gender dominance, which can fuel feelings of jealousy and insecurity. This is also consistent with a finding from a study done by Mercado and colleagues who noted that men beat their wives to assert control and realize their desires.^40^

Our study found that controlling behaviors by male partners was associated with attitude toward spousal physical abuse, which addressed our last research question. Controlling behaviors not only predispose women to various forms of violence, but it may also lead to indirect consequences for other members of the household. A previous study has highlighted the negative impacts of a husband/partner’s controlling behavior against a woman on the cognitive development of her children.^42^ Therefore, it is necessary to investigate factors that place women at risk of experiencing controlling behaviors by their husbands/partners. Research has shown that a wife is less likely to suffer from her husband/partner’s controlling behavior if she attains a higher level of education and income.^43^ This suggests that women’s socioeconomic characteristics may protect them from experiencing controlling behavior. Also, women’s attitudes toward spousal physical abuse could increase the experiences of controlling behavior by encouraging the husband/partner to exert more dominance. Therefore, research on the reciprocal effects of controlling behavior and attitude toward spousal physical abuse among women is necessary. Perhaps, future studies should concentrate on strategies for encouraging a zero-tolerance policy against spousal physical abuse while assisting in the transformation of societal paternalistic beliefs.

## Limitation

There are certain limitations that must be considered when interpreting the results of this study. The outcome and exposure variables used in this study were self-reported, which could lead to information or recall bias. The outcome of the study could have been underreported by study participants. This could be attributed to social stigmas associated with IPV, privacy concerns, the sensitive nature of the topic,^30^ not wanting the perpetrator to get in trouble, and fear of retaliation. Additionally, given the cross-sectional design of our study, causal conclusions on the effect of certain variables on the attitude toward spousal physical abuse cannot be drawn. There is potential for the existence of a bi-directional relationship between variables such as controlling behavior and attitude toward spousal physical abuse. Despite these limitations, the present study makes a valuable contribution to the existing body of global literature by focusing on the examination of factors that are correlated with spousal physical abuse.

## Conclusion

Women’s attitude toward spousal physical abuse plays a significant role in their risk of experiencing IPV. Further research is warranted to understand fully the mechanisms of association between partner-related characteristics and spousal physical abuse within the context of this study population. Future public health research should prioritize the investigation of messages, behaviors, and elements at system levels that have the potential to effectively address the social perspective of women about IPV in a positive manner.

## Data Availability

Data are available at https://dhsprogram.com/data/available-datasets.cfm

## Abbreviations Used

AOR: Adjusted Odds Ratio
CI: Confidence Interval
IPV: Intimate Partner Violence
LR: Logistic Regression
OR: Odds Ratio
P-NDHS: The Philippines National Demographic and Health Survey
VAW: Violence Against Women
WHO: World Health Organization
